# Mothers’ Experience of Social Change and Individualistic Parenting Goals Over Two Generations in Urban China

**DOI:** 10.3389/fpsyg.2021.487039

**Published:** 2022-01-03

**Authors:** Qinglin Bian, Yuyan Chen, Patricia M. Greenfield, Qinyi Yuan

**Affiliations:** Department of Psychology, University of California, Los Angeles, Los Angeles, CA, United States

**Keywords:** social change, China, culture, parenting goals, socialization, individualism, mothers, cultural values

## Abstract

During the past four decades, China has gone through rapid urbanization and modernization. As people adapt to dramatic sociodemographic shifts from rural communities to urban centers and as economic level rises, individualistic cultural values in China have increased. Meanwhile, parent and child behavior in early childhood has also evolved accordingly to match a more individualistic society. This mixed-method study investigated how social change in China may have impacted parenting goals and child development in middle childhood, as seen through the eyes of the current generation of mothers. Thirty mothers of fifth-grade elementary school students from Shenzhen, China were recruited and took part in semi-structured interviews. Participants answered questions and provided examples about their children’s life, their own childhood, and the perceived differences between the two generations. Participating mothers were also asked to rate which generation, themselves or their parents, cared more about the childrearing goals of academic competitiveness and socioemotional well-being. Using both qualitative and quantitative analysis, we expected and found an intergenerational increase in the perceived value mothers placed on individualistic traits: current mothers care more about their children’s academic competitiveness, personal happiness, and social adjustment, compared to their experience of their own mothers’ attitudes during their childhood a generation earlier. They also experience conflict between their children’s academic competitiveness and socioemotional well-being. As a function of both urbanization and increased economic means, children’s collectivistic family responsibilities for essential household chores have declined as the importance of schoolwork has increased.

## Introduction

Mothering is a set of cultural practices that reflect values and norms consistent with and adaptive to the surrounding ecology. When that ecology shifts, cultural values and parenting behaviors also shift, adapting to the new ecology (Greenfield et al., 2003a; Manago, 2012; Garcia et al., 2017). Our mixed-method study explores shifting mothering behaviors and values under conditions of social change in China.

Two basic value orientations—collectivism and individualism—define cultures (Greenfield and Bruner, 1966; Sampson, 1977; Ho, 1979; Hofstede, 1980; Bond et al., 1982; Triandis et al., 1986). These value orientations are reflected in mothering goals. Mothering in collectivistic cultures prioritizes social harmony and family needs, whereas mothering in individualistic cultures prioritizes individual independence and personal development (Greenfield et al., 2003a). These culturally structured child development goals guide mothering behavior; Harkness and Super (1996) include mothers’ childrearing goals in their concept of parental ethnotheories.

Another characteristic of culture is its instability, such that it can change along with changes happening in the sociodemographic environment. Greenfield’s multilevel theory of social change, cultural evolution, and human development places sociodemographic variables at the top level of the causal chain (Greenfield, 2009, 2016, 2018). Based on Tönnies (1957), two ideal sociodemographic ecologies are posited, *Gemeinschaft* (community) and *Gesellschaft* (society). Gemeinschaft ecologies are rural, agricultural, low income, and emphasize informal education at home. Gesellschaft ecologies are urban, commercial, relatively high income, and emphasize formal education at school. These variables operate both synergistically and individually; they influence both cultural values and learning environments. Therefore, when one or more sociodemographic variables change, so too do cultural values and learning environments. Changed learning environments in turn lead to shifting patterns of child and adolescent behavior (Manago, 2012; Weinstock et al., 2014; Garcia et al., 2015, 2017; Maynard et al., 2015; Abu Aleon et al., 2019).

Global sociodemographic shifts include increasing urbanization, rising income, rising education, and movement away from agriculture – a movement from more Gemeinschaft ecologies to more Gesellschaft ecologies. Across the world, these sociodemographic changes influence culture to shift from more collectivistic to more individualistic in both values and practices.

Note that we are talking about two different levels of analysis – Gemeinschaft and Gesellschaft constitute the sociodemographic level; collectivism and individualism constitute the cultural level. The levels are not identical; instead the basic relationship between them is one of adaptation: collectivism is conceived as adaptive in a Gemeinschaft ecology; individualism is conceived as adaptive in a Gesellschaft environment. Because of this connection, there is a causal connection between the levels: shifting ecologies produce cultural shifts. Therefore, the worldwide global shift in the Gesellschaft direction is posited to produce more individualistic values and practices. The much less frequent shifts in the Gemeinschaft direction move culture in the collectivistic direction (Park et al., 2014; Evers et al., 2021; Greenfield et al., 2021).

Strong empirical support for these theoretical propositions comes from Santos et al. (2017). Across 51 years of data (1960–2011) and 78 countries, individualistic values (desire for independent children, and preference for self-expression) and individualistic practices (living alone, high divorce rates, and small household size) were on the rise. Across the same 78 countries, sociodemographic factors of education level, income, urbanization, and movement away from agricultural work were intercorrelated, providing evidence of synergism. As a group, these sociodemographic variables predicted levels of individualistic practices 10 years later. Individualistic practices were intercorrelated and could be validly treated as facets of a single larger concept. Across the world, these same sociodemographic factors also predicted more individualistic values (friends more important than family, desire for independent children, and preference for self-expression) 10 years later. Like individualistic practices, individualistic values were intercorrelated and could therefore be treated as facets of a single larger concept.

But sociodemographic shifts not only produce cultural change; they also produce culturally consonant changes in children’s learning environments, the focus of the present research. For example, in Baja California, Mexico, decades of movement away from agriculture and the growth of urbanization were associated with a cross-temporal change in childrearing practices: mothers observed in 2015 set higher goals for their children’s achievement in an experimental game than mothers observed in 1972, 42 years earlier (Garcia et al., 2017). In a Gemeinschaft environment, children’s learning environment revolves around what Lancy (1996, 2016) calls the “chore curriculum.” These chores are contributions to family maintenance; they enact the collectivistic value of contributing to the family.

But not only children’s tasks, but also the way they are taught shifts to reflect new values. In Chiapas, Mexico, there was an intergenerational shift in the way Maya girls learned to weave as the community moved away from agriculture: in the early 1990s, mothers or other family members provided less help to learners than had occurred in the prior generation observed in 1970; in this way, the weaving “teachers” created a more independent learning environment for the learners (Greenfield et al., 2003b). In both cases, the shifts in learning environments implied greater encouragement of individual achievement or independence in their children.

In Bedouin communities in Israel, a rising level of parental education across the generations was associated with a shift in parenting goals: independence for girls took on greater value (Abu Aleon et al., 2019). Among Maya women in Mexico, movement to an urban environment and intergenerational increase in education were associated with more independent childrearing goals (Manago and Greenfield, 2011). The present study focuses on whether mothers in China, a rapidly changing society, construct new childrearing values and practices that reflect adaptation to rapid social change.

### Sociodemographic Change in China

In the past four decades, China has gone through rapid urbanization and modernization. The launch of economic reform brought nation-wide increase in wealth and dramatic sociodemographic changes from a rural, community-based, subsistence-farming ecology to an urban, market-oriented ecology (Yan, 2009; Bai et al., 2014; Zeng and Greenfield, 2015).

Sociodemographic change in China is reflected in the following statistics: from 1978 to 2016, there was an increase in urban residence from 17.9% to 57.4%. In these four decades, urbanization led to an increasing number of cities, and people were attracted to migrate from rural to urban areas. Education level has risen sharply: there were only 85.6 college students per 10,000 people in 1978; by 2016, the number had increased to 2695.8 college students per 10,000. Income has also increased: GDP per capita was 385 Yuan in 1978; by 2016, it increased to 53,980 Yuan (National Bureau of Statistics of China [NBSC], 2017). This time window from the 1970s to 2016 is relevant to the present study of mothers’ experience of intergenerational social change because, on average, participating mothers were born in 1975; all were interviewed in 2015. Our participant sample reflects these social changes. Our results are therefore applicable to urban populations with these sociodemographic characteristics; they would not be applicable to rural populations with low income and educational levels.

### Changing Cultural Values, Family Structure, and Childrearing Practices in China

Consistent with Greenfield (2009, 2016, 2018) theory of social change, culture, and human development and Ho’s (1989) prediction, the increase in urban residence, education level, income, and divorce rate, and the decrease in family size were closely related to a rise in individualism in China (Sun and Ryder, 2016). Individualistic words such as “choose” and “autonomy” became increasingly frequent in Chinese books from 1970 to 2008 (Zeng and Greenfield, 2015). However, collectivism has not declined as fast as individualism has increased. Certain collectivistic words, such as “obliged” have even become more frequent since the beginning of the market economy (Zeng and Greenfield, 2015). The present study also documents this reactive direction of social change, toward the intensification of certain collectivistic practices.

At the population level, certain individualistic practices, such as divorce, increased in this new ecology. Divorce is individualistic because it reflects the emphasis on personal happiness and the independence of each individual, as opposed to family stability (e.g., Manago et al., 2014). In China, the divorce rate has increased tenfold in this one-child policy era: in 1985, only 45.79 out of 10,000 couples were divorced, and the crude divorce rate was 0.44‰, whereas in 2016, 415.82 out of 10,000 couples went through divorce, and the crude divorce rate increased to 3.02‰ (National Bureau of Statistics of China [NBSC], 2017).

Most importantly in terms of parenting practices, family size decreased: in the 1950s, average families had five to six children; in 2015, the year our data were collected, average families had one or two children (O’Neill, 2021). This decrease was a reflection of the one-child policy which began in the 1970s. An intergenerational decline in family size was therefore expected in the sample used in this study.

A single child means that parents can focus heavily on the welfare and development of the one child (Feng et al., 2014). Research has shown that, in many ways, Chinese parents do focus their attention more on an only child than on a child with one or more siblings. Mothers and fathers of only children in Beijing reported spending more leisure time with their children than did parents of children with one or more siblings (Chen, 1986). In Hubei Province, more only-children reported that their mother and their father frequently played with them than did children with one or more sibling (Feng, 1993). So one-child families, on the whole, display more child-centered parenting (Ho, 1989). In response, only children are, at least in their teachers’ eyes, more self-centered than children with one or more siblings (Cunren et al., 1994).

Ecological and cultural change has shifted the ethnotheory of parenting in Chinese culture. Traditional Chinese parents were often described as strict, controlling, and critical of their children (Chen et al., 1998). Mothers and fathers held collectivistic values such as obedience, respect for authority and elders, perseverance, and academic excellence (Fong, 2007; Way et al., 2013). Traditional mothers were also perceived as less affectionate (Chao, 1994). Current Chinese mothers prioritize individualistic values such as personal achievement, autonomy, materialism, freedom of choice, and formal education (Liu et al., 2005). In order to adapt to this new market-oriented environment filled with both opportunity and competition, individualistic skills such as autonomy, self-expression, and assertiveness have become more important to survival and success, especially in urban contexts. Chinese parents with higher educational levels, typical of a Gesellschaft ecology, have adolescent children with more individualistic or independent emotions, such as pride and a sense of superiority. Chinese parents with lower educational levels, typical in a Gemeinschaft ecology, have children with more collectivistic or interdependent emotions, such as respect and closeness (Hamamura et al., 2013). Desirable traits in individualistic, Western contexts such as self-confidence and extraversion have come to be valued more, while traditionally desirable traits in Chinese culture, such as shyness, have become less accepted (Chen et al., 2005).

### Intergenerational Change in China

Shifting cultural values and shifting parenting styles are reflected in intergenerational value change. Younger generations in China are more individualistic than older generations in their values and behaviors (Sun and Wang, 2010). Relevant to our study, people in younger generations are more vocal and open about intimate thoughts and feelings (Yan, 2003). Lee (2011) has observed an expansion of norms for self-expression.

In a qualitative study, Way et al. (2013) found that the foremost concern of Chinese mothers of seventh graders was the happiness and mental health of their children; they were also very concerned about their children’s social skills. Could these concerns represent a generational shift? Xu and Hamamura (2014) provide an affirmative answer to this question; they found that a national sample of adults surveyed online considered that individual happiness and psychological health had become more important to Chinese people in the last 50 years. In the present study, we predicted that this perception of a generational shift would also be experienced by current Chinese mothers in comparing how they were raised with how they were raising their children. Indeed, the mothers in Way et al.’s qualitative study perceived that how they were raised was no longer relevant, and they reported that they allowed their children more autonomy than they had been given as children.

Zhou interviewed Chinese grandmothers to learn about their experiences of social change as it impacted parenting practices and child behavior across three generations in their own families (Zhou et al., 2017). Grandmothers were asked to compare childhood experiences and parenting experiences in their own generation, their children’s generation, and their grandchildren’s generation. Compared with earlier generations, grandmothers observed that the current generation of young children exhibit the strongest individualistic traits, such as autonomy and curiosity, and the weakest collectivistic traits, such as obedience and shyness. They perceived that the learning environment had changed over the generations in a corresponding fashion: later generations of parents were perceived to exhibit child rearing behaviors that foster individualism in children: higher levels of parental support and praise combined with lower levels of criticism. In accord with Greenfield’s theory, a statistical model showed that these intergenerational changes in perceived parent behavior were linked to intergenerational changes in perceived child behavior. This research furnishes an important foundation for the current study.

### Moving From Country to City in China

Part of the intergenerational shift toward being more vocal about intimate thoughts and feelings, relates to urbanization as major shift for much of the Chinese population. Potter (1988) in his ethnographic study of rural Chinese social life characterizes the cultural construction of emotion that he found in the village in this way: “Attention is directed away from the psychological processes of individuals, especially their feelings, and toward the appropriate expression of shared intersubjective agreement about …the social world” (p. 191).

It is therefore noteworthy that most past research on parenting in China has focused on urban populations. As one would expect from Greenfield’s theory of social change, culture, and human development, urban populations in China are more individualistic than rural (Cai et al., 2012). In terms of child rearing, they encourage greater independence in their adolescent children and are less controlling (Chen et al., 2010). So one would expect that migration from rural to urban areas would alter parenting values and practices in an individualistic direction.

Chen and Li (2012) studied Chinese mothers and fathers who had farmed at the outskirts of the city but were reclassified as urban residents because of urban expansion. This reclassification meant that rural residents gave up their right to use the land, but obtained the same rights to welfare benefits and employment opportunities that regular urban residents enjoy. Indeed, their parenting behavior became similar to parents who had always lived in the city and differed from parenting behavior of regular rural residents: like the regular urban residents, they reported that, as parents, they encouraged their children to take initiative in activities, express opinions, and be independent – the whole suite of individualistic child behaviors.

But we expected losses as well as gains in children’s learning environment. We thought that the rural-urban shift would lead to the loss of subsistence activities such as farming, a major part of informal education in rural contexts (e.g., Greenfield, 2004) and therefore a loss in children’s participation in and learning from subsistence tasks. That is, we expected the collectivistic “chore curriculum” (Lancy, 1996, 2016), typical of a learning environment adapted to Gemeinschaft conditions, to be reduced. These chores relate to maintaining family subsistence.

### Challenges and Potential Conflicts in Current Chinese Parenting

As individualistic values become more important in China, current mothers in urban China are becoming more concerned with their children’s academic achievement; they expect their children to be competitive in school in order to prepare for future personal success (Way et al., 2013). Academic achievement is adaptive to compete successfully in the new market-oriented environment. In line with grandmothers’ perceptions (Zhou et al., 2017), Chinese mothers may therefore have become more controlling of their children’s academics. They may pay more attention to their children’s grades and class ranking, and they may impose more academic guidance on their children’s academic life. This is Gemeinschaft-adapted or collectivistic parenting for the individualistic goal of academic competitiveness.

On the other hand, current Chinese mothers may also have a growing concern for their children’s mental well-being and social adjustment. Concern over the psychological well-being of the individual is another facet of individualism (Steele and Lynch, 2013). Social and emotional well-being prioritizes personal happiness and promotes the focus on one’s inner feelings; it is less prioritized in Gemeinschaft environments, which emphasize action rather than feeling (Greenfield, 2013).

Self-focused emotion, that is, focus on one’s inner feelings, also contrasts with other-focused emotion. Other-focused emotions (e.g., empathy) serve collectivistic goals of social belonging and harmony and are adaptive in Gemeinschaft environments. Self-focused emotions (e.g., authentic expression of negative emotion) serve individualistic goals of self-assertion and are adaptive in Gesellschaft environments. Chinese mothers have traditionally socialized children to be aware of other people’s feelings rather than their own (Trommsdorf and Heikamp, 2013). Wang (2003) found this to be the case for Chinese mothers. This was particularly the case in rural areas.

However, focus on a child’s own feelings is intrinsic to concern with a child’s social-emotional well-being. Hence movement from both other-focus and action to self-focus and inner feelings would lead to an increase over time in maternal concern with social-emotional well-being.

However, the two individualistic goals: academic competitiveness and social-emotional well-being can be in conflict. The maternal requirement of staying competitive at school may bring stress to the children, and it requires extra time after school to work on academic materials in order to acquire more advanced knowledge. These academic achievement demands can sacrifice children’s time to play and interact with peers; this time is essential for developing social skills and maintaining emotional well-being. Indeed, today’s Chinese mothers of middle schoolers reported that “a common impediment to happiness and health… was the amount of academic pressure placed on their children” (Way et al., 2013, p. 65).

Current Chinese mothers therefore face the challenge of balancing the two individualistic goals. In the present research we ask: is this challenge a generational shift?

Chinese mothers may also have less experience and knowledge than parents in the United States of parenting practices that are well adapted to an individualistic society. When the current generation of parents were in their childhood, China was more collectivistic, and their parents might have raised them in a way that was well adapted to the collectivistic culture. Their lack of personal experience with individualistic parenting may make it difficult for them to raise their children in an increasingly individualistic culture.

Basic to this issue is whether or not today’s Chinese mothers perceive that the way they were raised can function as a guide to raising their own children. Qualitative interviews revealed that Chinese mothers do not: they felt that how they were raised was no longer relevant in raising their own children (Way et al., 2013). But what exactly are the perceived intergenerational differences in parenting? This was our basic research question, one that we explored both quantitatively and qualitatively. In this research, we focus on mothers rather than fathers because mothers do most of the childrearing in China. Despite greater involvement of fathers with childrearing compared with earlier times, “Chinese fathers still lag behind mothers in the amount of time they invest in child care” (Li, 2020, p. 153).

### The Present Research

#### Intergenerational Sociodemographic Changes

Based on the sociodemographic changes in China as a whole, we formulated the following hypotheses about intergenerational change in our sample:

**Hypothesis 1:** *More children than mothers are growing up in an urban environment.*

**Hypothesis 2:** *Family size will decrease from the mothers’ to the children’s generation.*

**Hypothesis 3:** *Mothers will have more formal education than their mothers, the grandmothers, had.*

**Hypothesis 4:** *There will be an intergenerational shift toward professionalism in mothers’ work.*

**Hypothesis 5:** *There will be an increase in divorce across the generations.*

#### Intergenerational Shifts in Mothers’ Childrearing Goals and Practices

We thought that these intergenerational sociodemographic shifts would lead to intergenerational shifts in mothers’ childrearing goals and practices. Our hypotheses and question follow:

**Hypothesis 6:** *Today’s mothers will have more individualistic and less collectivistic goals for their children than their mothers had for them.* This hypothesis was based on Greenfield’s theory and the research described in the introduction.

**Hypothesis 7:** *Today’s mothers of young children will perceive that their children’s grades and academic competitiveness are more important to them than they were to their own mothers.* As early as 1986, Yang (1986) concluded “that social-oriented achievement motivation is declining in Chinese society in the process of modernization, whereas individual-oriented achievement motivation is gaining ascendancy” (Ho, 1994, p. 295). Grades and academic competitiveness lie at the center of individual-oriented achievement.

**Hypothesis 8:** *Today’s mothers of young children will impose more parental guidance and control on their fifth-grade children’s academic work and activities*. This hypothesis was based on the idea that current Chinese mothers have intensified some collectivistic childrearing behaviors in order to deal with the current greater emphasis on children’s educational achievement. Zhou et al. (2017) had found intergenerational increase in parental control for children between 4 and 6; the present study hypothesized that this historical trend would extend developmentally to older children.

**Hypothesis 9**: *Today’s mothers of young children will perceive that they are more concerned about their children’s psychological and social well-being than was the case for their mothers.* Way et al.’s (2013) qualitative study indicated that mothers in China were very concerned about their children’s happiness, mental health, and social skills, and mothers recognized that the way they were raised was no longer relevant. However, they did not specifically identify concern for children’s well-being as an intergenerational change. Their very interesting study was qualitative. To our knowledge, we are the first to do a quantitative analysis and the first to explicitly focus on the intergenerational increase in Chinese mothers’ concern for their children psychological well-being.

#### Comparing Conflict Across the Generations

The issue of conflicting goals was addressed through one hypothesis and one question:

**Hypothesis 10:** *Today’s mothers of young children will perceive themselves as experiencing more conflict, compared with their own mothers, between two developmental goals for their children: academic competitiveness and social-emotional well-being.* The mental health costs of school pressure have been known since the 1980s (Cheung, 1986). However, until recently they were not explored from mothers’ point of view. Way et al. (2013) identified this conflict in their qualitative interview study of Chinese mothers. To our knowledge, we are the first to explore the maternal perspective on this issue as a matter of intergenerational change.

#### Intergenerational Shifts in Children’s Activities

**Hypothesis 11:** In adapting to more Gesellschaft environments in the current generation (e.g., urbanization, more technology, greater economic resources), children will lose activities that contribute to family subsistence; these tasks will be replaced by school-related activities such as homework. This is an issue that is highlighted in an indigenous Maya community in Mexico (Maynard et al., in revision), but has not been explored in China. We also explored whether meaningful contributions to the household would decline across the two generations in favor of schoolwork even when mothers were raised in the city. Meaningful contributions to the household are considered an important manifestation of family-centered collectivism. This hypothesis is addressed by means of qualitative analysis.

**Question:** *How do mothers experience shifting developmental goals and navigate conflict between them?* This question is answered in the qualitative analysis.

In order to explore these hypotheses and question, we designed a mixed-method study, described in the next section.

## Materials and Methods

### Setting

The study was conducted in Shenzhen, China in 2015. Shenzhen is a metropolis in southern China of close to 11.4 million people (as of the end of 2015) (Shenzhen Government Online, 2017). Growing from a little village relying on fishing 30 years ago, the city of Shenzhen has developed into a modernized international metropolis (Ni, 2016).

### Participants

Thirty Chinese mothers of Grade 5 elementary school children participated in this study. The reason for selecting fifth grade for the study was the possibility of additional academic or other extracurricular activities at this age, activities that point to a child-centered achievement focus on the part of the mothers. Fifth grade is also an important phase for childrearing, when academic pressure is building up from students graduating from elementary school in 1 year and competing for the entrance to a good middle school.

The mean age of the mothers was 40 (*M* = 39.77, age range: 35–46 years, *SD* = 3.34) and the mean age of their children was 11 (*M* = 10.93, age range: 10–12 years, *SD* = 0.52). In 2015, participants were recruited from a public elementary school in Shenzhen. Participants were homogenous in that they all have the same mainstream Chinese ethnicity, and all are from a middle- to upper-middle-class background. Our earlier study comparing the perceived behavior of Chinese parents across three generations was the basis for concluding that 30 mothers would be sufficient to reveal statistically significant intergenerational change.

Social-class background was partly ascertained by the school’s neighborhood and the SES requirements for attending it. The school is located in one of the more affluent districts in Shenzhen. The neighborhood is located about one or two miles away from the city center (Shenzhen Government, library, music hall, etc.). In order to attend this school, parents need to own a property in that neighborhood (generally an apartment, as there are no houses nearby). Having parents who rent a place or simply reside in the neighborhood does not qualify children for the school. Mothers also estimated their annual family income; the mean estimate, based on 26 of the 30 mothers, was 369,231 Chinese Yuan (58,079 U.S. dollars at the time of the study).

Six of the mothers had lived in a rural area when they were their children’s age. Nine had lived in a suburban area. In China, suburban areas serve as a transition when residents from the rural areas migrate to urban centers. Fifteen mothers, that is, half the sample, lived in an urban environment when they were their children’s age.

Although the question was not asked individually, ethnographic observation by the second author indicated that grandparents living with the nuclear family is a very common practice, especially when both parents are working full time. The grandparents are most likely to stay with the nuclear family during summer breaks (early July to September 1st), because parents are working and they need someone to look after the children. If we had used grandparents as informants, some would therefore not have had contact with the children during the school months, our focus of interest. In addition, our point of access was the fifth-grade teacher. In fifth grade, teachers have access to parents but not to grandparents.

### Positionality of the Researchers

QB, YC, and QY grew up in China and are native Mandarin Chinese speakers. QB and YC lived in China until their college years, when they came to the United States. QY lived in China until high school when she came to live in the United States. All of these native Chinese co-authors travel back to China for periodic visits. The second author and interviewer YC is from Shenzhen where the interviews were carried out; she has family connections to the school where the research took place. All data analysis and interpretation were done by the three Chinese authors. PG is a cultural and cross-cultural researcher who has previously worked in collaborations led by two sets of Chinese researchers (Zeng and Greenfield, 2015; Zhou et al., 2017).

### Interview Instrument

Participants were asked to report their perceptions of the parenting practices and values of two generations. The first generation refers to mothers’ own childhood experiences of how they were parented. This generation reflects the outcomes of child rearing strategies and parenting goals during the beginning stage of a market economy (in the 1980s). The second generation refers to mothers’ reports of parenting their own children and their impressions of their own children’s experiences of being parented. This generation reflects the current child rearing strategies and parenting goals, as the market economy is maturing.

Each interview consisted of two parts: first, mothers provided examples and ranked the importance of academic competitiveness and socioemotional well-being in their own childhood (first generation) compared with their children’s generation (second generation). Mothers were also asked to provide examples of conflicts between the two constructs in both generations. During the interview, mothers discussed differences they perceived in parenting goals in their childhood and their child’s current life. Responsibility for household chores in their childhood and their children’s often came up spontaneously in the conversation. Lastly, mothers provided their family’s sociodemographic information for two generations, including education level, occupation, and degree of urbanization.

### Mixed-Method Design: Relationship Between Qualitative and Quantitative Data

In our main research design, qualitative data are embedded in a quantitative framework. Ranking of a number of variables provided the basic quantitative data for the study. These variables are shown in Table 1. Interview questions, shown in the table and the Supplementary Appendix, were a conversational way to elicit these rankings. The questions also produced qualitative data used in our case studies.

**TABLE 1:** Coding categories and examples.

A. Coding categories and examples: Goals and relationship between goals of academic competitiveness, health, and social/emotional well-being.		
For each variable, footnotes present the eliciting question or questions.	Generation 2 mothers perceived conflict	Generation 2 mothers did not perceive conflict
**Explicit perceived conflict between academic competitiveness and social/emotional well-being^6^**	“There is some [conflict between academic competitiveness and his psychological well-being] …. He did not want to, but he would still do work outside of school.”	“There is not much pressure from the school, so my child did not have this type of conflict.”

	No implicit conflict in either generation	Implicit conflict in Generation 1 children only	Implicit conflict in Generation 2 children only
**Implicit conflict between academic competitiveness and social/emotional well-being^7^**	“I think parents at that time might care about children’s academics more… My parents paid relatively less attention [to my happiness], they looked at your grades more….[My child’s happiness is] quite important….[My child’s social adjustment is] very important.”	(This category never occurred.)	“I think [my child’s academics] should be quite important… I pay attention to my child’s emotions… I think [my child’s social adjustment] is very important… My requirement for my child [about academics] is much stronger than that of my parents….[My parents] thought that happiness was not that important….[They paid] little attention [to my social adjustment].”

	Academic achievement is not important at all		Health and well-being of the child is equally important or more important than academic achievement.	Academic achievement is more important than the health and well-being of the child
**Relative importance of academic competitiveness versus health and social/emotional well-being^8^**	“… At that time before, what we have called it, um, focusing mainly on food… my parents were all about survival, they…. they would just let you receive formal education.”		“I will pay attention to both [academics and well-being], do not break the balance.”	“She [participant’s mother] did not think that emotions or psychological awareness was an important issue at all. She felt that the only thing that was the most important was [whether] your grades reached [her standards] or not.”

	More collectivistic for Generation 1 mothers	More individualistic for Generation 1 mothers	Both generations the same	More individualistic for Generation 2 mothers
**Goals^9^**	“I think [my child] should at least be a person who has a skill [to afford her living]….[During my childhood,]….[My] parents just said you should study hard, listen to your teachers, and we did not have any after-school classes.”	“[My goal for my son is] to be an honest and responsible person, … useful to the society…. [My mother expected me to] have better grades as a girl and have something I want to pursue.”	“I feel like my parents had the same expectation for me [as my expectation for my child]: hope the child to be happy and discover fun when they grow up.”	“Definitely I have more expectations for my son. At least I would like him to do what he wants to do… At that time my parents showed no expectations for me, or at least did not reveal any.”

For each variable, footnotes present the eliciting question or questions.	More or more important to Generation 1 mothers	Both generations the same	More or more important to Generation 2 mothers
B. Coding categories and examples: Generational ranking of academic achievement, grades/class rank, academic guidance/control, happiness, and social adjustment.			
**Academic achievement^1^**	“I feel like parents probably cared more about the academics of their children at that time.”	“I [cared] for my daughter about the same as my parents did for me.”	“[My academic achievement and competitiveness] were not important to my parents at all… We care more now.”
**Grades and class rank^2^**	“[She] forced her expectations onto me… and required me to get 100 points, not even 99 or 98… so I would not expect much for my child.”	“I feel like my mother’s requirements were the same as what we have for our child.”	“I have much stronger requirements than my parents did. I expect my son to be the top five in the class in everything.”
**Academic guidance and control^3^**	“She had this behavior everyday: looking at homework, checking your schoolbag, pencil case, nothing would escape [her]… Besides the usual tutoring classes, my child plans for herself pretty well … She can make pretty comprehensive plans for herself; she does not listen to my plans.”	“Sometimes I got annoyed when I was doing my homework and did not want to finish it, my mother would talk to us in similar ways, and I learnt it from her.”	“…at that time, we went to school as required, and our parents never managed our homework or other school stuff… When it comes to his [my son’s] academics, … to be honest I am constantly watching him…. Yes, sometimes I also feel sorry for him to be controlled this much…”
**Happiness^4^**	(This category never occurred.)	“[The happiness of our daughter] is definitely very important to us…. I feel like my parents probably cared a lot about our psychological well-being.”	“We care a lot about our son’s happiness… My parents never cared this much.”
**Social adjustment^5^**	(This category never occurred.)	(This category never occurred.)	“I asked about my son’s social adjustment… My parents didn’t care much about this.”

*^1^Q3: How important is your child’s academic achievement and competitiveness? Q4: Do you think your academic achievement and competitiveness was more or less important for your mother when you were a child, or was it the same?*

*^2^There was not an additional question on this topic. However, information often came up spontaneously in response to Q3 and Q4 above.*

*^3^There was not an additional question on this topic. However, information often came up spontaneously in response to Q3 and Q4 above. Related responses also showed up in: Q1: What are your goals for your child? (or what do you want your child to be like when he/she grows up? Q2: Are these similar or different from your mother’s goals for you when you were a child? If different, how? Q9: Do you ever feel that there is a conflict between your child’s academic competitiveness and his/her social/emotional well-being? Why? Why not? Q10: Do you think your mother ever felt that there was a conflict between your academic achievement and your social/emotional well-being? Why? Why not? Q11: What after school activities does your child engage in? Q12: Who selected the activities? Why? Q13: When you were about the age of your child, what after-school activities did you engage in? Who selected the activities? Why?*

*^4^Q5: How important is your child’s happiness? Q6: Do you think your happiness when you were a child was more or less important to your mother, or was it the same?*

*^5^Q7: How important is your child’s social adjustment to you? Q8: Do you think your social adjustment when you were a child was more or less important for your mother, or was it the same?*

*^6^Questions on this topic: Q9: Do you ever feel that there is a conflict between your child’s academic competitiveness and his/her social/emotional well-being? Why? Why not?*

*^7^Questions on this topic: Q3: How important is your child’s academic achievement and competitiveness? Q4: Do you think your academic achievement and competitiveness was more or less important for your mother when you were a child, or was it the same? Q5: How important is your child’s happiness? Q6: Do you think your happiness when you were a child was more or less important to your mother, or was it the same? Q7: How important is your child’s social adjustment to you? Q8: Do you think your social adjustment when you were a child was more or less important for your mother, or was it the same?*

*^8^There was not an additional question on this topic. However, information often came up spontaneously in response to the following questions: Q1: What are your goals for your child? or what do you want your child to be like when he/she grows up? Q2: Are these similar or different from your mother’s goals for you when you were a child? If different, how? Q3: How important is your child’s academic achievement and competitiveness? Q4: Do you think your academic achievement and competitiveness was more or less important for your mother when you were a child, or was it the same? Q5: How important is your child’s happiness? Q6: Do you think your happiness when you were a child was more or less important to your mother, or was it the same? Q7: How important is your child’s social adjustment to you? Q8: Do you think your social adjustment when you were a child was more or less important for your mother, or was it the same? Q9: Do you ever feel that there is a conflict between your child’s academic competitiveness and his/her social/emotional well-being? Why? Why not? Q10: Do you think your mother ever felt that there was a conflict between your academic achievement and your social/emotional well-being? Why? Why not?*

*^9^Questions on this topic: Q1: What are your goals for your child? (or what do you want your child to be like when he/she grows up?) Q2: Are these similar or different from your mother’s goals for you when you were a child? If different, how?*

Embedding is one of the mixed-method designs identified by Creswell and Clark (2007); in this design, qualitative data support the quantitative findings. In the present study, qualitative examples illuminate how quantitative shifts in perceived parenting goals over two generations were experienced by our sample of Chinese mothers.

### Qualitative Analysis

For the issue of rural-urban migration, where the relevant sample size was very small – limited to six mothers who had grown up in rural areas and then moved to the city – we simply present qualitative examples. Chores are the dependent variable that was analyzed in relationship to rural-urban migration. We also present a qualitative analysis of chores to show intergenerational change, even within the city.

### Procedure

An announcement of the study was posted on an online chat group that was used for communications between teachers and parents of Grade five students at the elementary school. Semi-structured interviews were conducted with 30 Chinese mothers individually. All interviews were conducted in Mandarin Chinese by the second author in a quiet office at the elementary school.

After each participant entered the office, the interviewer informed her of the purpose and confidentiality of the study. Each participant was asked to consent to the study by signing a consent form. Participants were informed that the interview would be recorded and that they could ask questions or withdraw from the study at any time. After the consent procedure, the interviewer began to ask questions and record the interview with a voice recorder. Each interview took from about 15 to 45 min, with an average of approximately 24 min. After the interview, each participant received a small gift, a pen with a UCLA logo (worth about $5) as compensation.

Each interview consisted of 14 questions about parenting in mother’s and grandmother’s generation. There were also 12 sociodemographic questions. The complete list of interview questions is presented in the Supplementary Appendix in both Chinese and English.

### Language and Translation

All interviews were conducted in Mandarin Chinese by YC, who is from Shenzhen and whose native language is Mandarin. Interview questions were developed in both Chinese and English by YC, under the guidance of the PG, whose native language is English. All recordings were transcribed verbatim by QB, a native Mandarin Chinese speaker. Finally, the translation from Chinese to English represented in the case studies in the qualitative analysis was completed by authors QB and QY.

### Variables and Coding

#### Parenting Goals and Intergenerational Comparison

YC asked mothers: “What are your goals for your child? (or what do you want your child to be like when he/she grows up?” and “Are these similar or different from your mother’s goals for you when you were a child? If different, how?” (These are Questions 1 and 2 in the Supplementary Appendix.) For both generations of mothers, responses were coded into four categories: “1” if the goals are more collectivistic or adapted to a Gemeinschaft ecology; “2” if the goals are more individualistic or adapted to a Gesellschaft ecology; we coded “1.5” if mothers have both types of goals; we coded “0” if mothers seem to have no goal or expectation for their children at all. In particular, we coded children’s doing subsistence chores, such as cooking and taking care of younger siblings, as collectivistic or Gemeinschaft-adapted goals. We coded expectation of qualities, such as upright, honest, and kind, as collectivistic goals. Also, if mothers had expectations of ascribed gender roles of their children, such as a girl should not be too “bossy,” as a Gemeinschaft-adapted goal. Individualistic or Gesellschaft-adapted goals for children included mental health, happiness, goal orientation, independence, and academic accomplishment (see Table 1A for examples).

These separate assessments of goals in the two generations were then transformed into ranks. If the second generation, i.e., the mother, perceived herself as more individualistic in her childrearing goals than her mother had been, the rank code was 2. If she perceived herself as more collectivistic in her childrearing goals than her own mother had been, the ranking was 1; if the participant perceived both generations as similar in their childrearing goals, the rank code was 1.5. If one generation was perceived as not having any childrearing goals, then perception of the other generation’s goals was used for the ranking. For example, if the participant perceived her mother as not having any childrearing goals, but she had collectivistic goals for her child, the rank score would be 1. If direct comparison was not made by the mother the coder made an inference based on relative number of collectivistic and individualistic goals.

#### Intergenerational Ranking of Other Variables

Participants were asked to rank the two generations of mothers – themselves as mothers and their own mothers – on the importance of academic achievement, academic guidance/control, parents caring about children’s grades, parents caring about their children’s happiness, and parents caring about their children’s social adjustment. There are three intergenerational ranking patterns for coding these variables. For example, “2” on academic achievement stands for the perception that mothers of the second generation (the current children) view academic achievement as more important than mothers of first generation (grandmothers of the current children). “1.5” stands for equal perceived importance of academic achievement in each generation, while “1” stands for the perception that the mothers’ own mothers (first generation mothers) view academic achievement as more important than mothers of second generation. The same coding scheme was used to compare and rank mothers’ experience of parental academic guidance/control, parents caring about grades, happiness, and social adjustment between their own mother (Generation 1) and themselves as mothers (Generation 2). Examples of these variables are found in Table 1B.

Mother’s perception of whether her child has experienced or is undergoing conflicts between achieving academic competitiveness and maintaining socioemotional well-being was coded for second-generation mothers only. We did not think mother could report on this for her own mother. Therefore, our method did not allow this variable to be assessed for the grandmothers.

We coded “1” if the mother perceived that her child had experienced/was experiencing conflicts, and we coded “0” if the mother perceives that her child has no conflict. This variable was called “explicit perceived conflict.” Please see the top of Table 1A for examples.

We also coded implicit conflict for children between their mothers’ academic goals and their mothers’ socioemotional goals. This was an intergenerational comparison and was achieved by comparing codes for academic competitiveness with codes for happiness and social adjustment for both generations. If one generation valued both academics and socioemotional well-being of their child more than the other generation, this situation would create potential tension in areas such as time management, which may eventually lead to greater or more frequent conflicts for the child when attempting to balancing academics, happiness and social adjustment. We coded implicit conflict as “0” if there was no implicit conflict perceived in either generation; “1” if mothers of the first generation valued their children’s academics more than the second generation and also valued their happiness and/or social adjustment more than the second generation (more implicit conflict perceived in Generation 1 children). We coded “2” if mothers of the second generation valued their children’s academics more than the first generation and also valued their happiness and/or social adjustment more than the first generation (more implicit conflict perceived in Generation 2 children). All instances fell into two categories: “no conflict perceived in either generation” (0) or “more conflict perceived in Generation 2 children” (2). Examples of these two categories are found in Table 1A.

In addition to ranking the two generations for intergenerational change in the presence of conflict between academic achievement and social development, we also coded each generation separately for which was more important for that generation, the child’s academic achievement or the child’s social development. This was not based on a particular question but often came out as part of the conversation. The coding categories for this variable were as follows:

• Academic achievement is not important at all.
• A child’s health and well-being are equally or more important than academic achievement.
• Academic achievement is more important than the health and well-being of the child.

Please see Table 1A for examples.

#### The Coding Process

Initial coding definitions were developed by Author YC. The coding system was refined through an iterative process of three-way communication among Authors QB, PG, and QY. These definitions were then incorporated by Author QB into a code book used for both master coding and reliability coding.

##### Reliability Coding

Except for the variable of Goals and of Relative Importance of Academic Competitiveness vs. Health and Social/Emotional Well-being, where the roles were reversed, the Author QB served as the master coder, and reliability coding was done by the Author QY. Both coders are native Mandarin speakers. After sufficient reliability was established between Authors QB and QY, the small number of disagreements was resolved through three-way discussion by Authors QB, PG, and QY.

Twelve interviews (40% of the total) were selected, and the reliability coder coded eight quantitative variables: Goals; Academic Achievement and Competitiveness; Parent Caring about Grades/Class Ranking; Parent Providing Academic Guidance/Control; Happiness; Social Adjustment; Perceived Conflict between Academic Achievement and Psychological Well-Being; and Implicit Conflicts. Whereas 20% of a sample is a common figure for reliability coding, we used 40% of the sample because of the small sample size (Syed and Nelson, 2015).

Cohen’s Weighted Kappa was used to compare scores between the original coder and the reliability coder. For Academic Achievement and Competitiveness, results reached almost perfect agreement (*k* = 0.83, *N* = 12); for Parental Academic Guidance/Control results also reached almost perfect agreement (*k* = 0.89, *N* = 12) (Landis and Koch, 1977). There was perfect agreement between the coders for all six of the other variables: Parenting Goals, Parent Caring about Grades/Class Ranking, Happiness, Social Adjustment, Perceived Conflicts, and Implicit Conflicts.

For importance of academic compared with social parenting goals, the mother’s views of her goals for her child and her views of her own mothers goals for her when she was a child were coded separately. For this variable, the Author QY was the main coder and the Author QB served as reliability coder. Twenty-four goal responses, half from each maternal generation, were coded by both coders; again, this was 40% of the sample. Kappa with linear weighting was 0.71; this is considered substantial agreement (Landis and Koch, 1977).

#### Quantitative and Qualitative Analyses

Because all the variables are categorical, we tested our quantitative hypotheses by means of non-parametric statistics: We used *X*^2^ for variables where there were separate judgments made for the two generations (Preacher, 2001) and binomial tests for ranking variables, where only a single judgment is made by the participant. Preliminary statistical analysis was done by the Author YC. Authors QB, PG, and QY carried out the final quantitative analyses used for this article.

For addressing the effect of urban vs. rural residence and generational change on children’s chores as a childrearing goal, our analysis is entirely qualitative, as this issue was not probed by any question, and so came up spontaneously in only a small subset of the interviews. Authors QB, YC and QY carried out the qualitative analyses.

## Results

### Intergenerational Sociodemographic Change

**Hypothesis 1:** *More children than mothers are growing up in an urban environment.* The demographic data reflect the nation-wide migration from country to city: Only 50% of the first generation (the mothers) lived in an urban area when they were nine to 13 years of age. The other 50% of the mothers lived in rural areas or suburban areas during childhood and migrated to Shenzhen, one of the biggest metropolises in current China, after they grew up. Twenty-four of the thirty children (the second generation) were born and raised in Shenzhen. The remaining six were born in various provinces, but they all moved to Shenzhen before school age. Therefore, all the children were spending middle childhood, the focus of this research, in an urban environment. Significantly more children than mothers spent their childhood in an urban environment (see Table 2A for statistical details).

**Hypothesis 2**: *Family size will decrease from the mothers’ to the children’s generation.* Family size decreased significantly from one generation to the next. Only 10% of the participant mothers were only children; 40% of these mothers had one sibling, and 50% had two or more siblings. However, the second-generation children have significantly fewer siblings: 83.3% of the children are only children, and the remaining 16.7% have only one sibling (see Table 2B for statistical details).

**Hypothesis 3:** *Mothers will have more formal education than their mothers had.* The grandmothers of the current children received less formal education than the mothers. Only 6.7% of the grandmothers had a bachelor’s degree or higher. However, when looking at the mothers’ education level for the second generation, 76.7% had a bachelor’s degree or higher (see Table 2C for statistical details).

**Hypothesis 4**: *There will be intergenerational shifts toward professionalism in mothers’ work.* Mothers’ careers also differed in the two generations. Twenty percent of the grandmothers were farmers, and 23.3% of them engaged in professional careers, such as in teaching and in engineering. In contrast, no current mothers have a career in agriculture; 80% of them are in professional occupations, such as doctors, teachers, financial managers, and businesswomen. The dominant category for the grandmothers (57%) was very diverse; it included self-identification as a “worker,” part-time jobs, and housewife; no mother used any of these terms to describe her work. The only terms that were used by a few members of each generation were “freelancer” and “company employee,” so there was little overlap between the two generations (see Table 2D for statistical details).

**Hypothesis 5:** *There will be an increase in divorce across the generations.* As in China as a whole, there was a small increase in divorce in the mothers’ generation. All grandmothers were married, and their children, the current mothers, were all raised in two-parent families. In the sample of 30 current mothers, there was one divorce. This is very similar to the contemporaneous divorce rate in China as a whole and a similar intergenerational increase.

**TABLE 2:** Intergenerational differences in sociodemographics.

A. Residence in middle childhood.						
	Urban	Rural/suburban	Chi-square	*df*	*p*	
Mothers	15	15	17.42	1	<0.001	
Children	30	0				

B. Family size.						
	Only child	1 sibling	2 or more siblings	Chi-square	*df*	*p*
Mothers	3	12	15	35.17	2	<0.001
Children	25	5	0			

C. Education.						
	Less than bachelor’s degree	Bachelor’s or higher	Chi-square	*df*	*p*
Grandmothers	28	2	30.24	1	<0.001
Mothers	7	23			

D. Careers.						
	Farmers	“Worker,” part-time work, housewife, etc.	Professional	Chi-square	*df*	*p*
Grandmothers	6	17	7	20.58	2	<0.001
Mothers	0	6	24			

### Intergenerational Shifts in Mothers’ Childrearing Goals and Practices

The intergenerational sociodemographic differences described above were expected to drive the changes in childrearing goals and parenting practices constructed by our participant mothers.

**Hypothesis 6:** *Today’s mothers will have more individualistic and less collectivistic goals for their children than their mothers had for them.* In 20 out of 30 cases, the mothers perceived that their mothers, the grandmothers of the children, had more collectivistic goals for their children, more accurately, goals adapted to a Gemeinschaft ecology, whereas today’s mothers, the participants, have more individualistic goals for their children, goals adapted to a Gesellschaft ecology (see Table 3 for statistical details).

**Hypothesis 7:** *Today’s mothers of young children will perceive that their children’s grades and academic competitiveness are more important to them than they were to their own mothers*. Comparing their own mothers with themselves as mothers, 24 out of 30 participant mothers perceived their children’s academic achievement to be more important to them than their own academic achievement had been to their mothers when they were children. Seventeen out of 27 participant mothers also perceived that they emphasized their children’s grades and class ranking more than their own mothers had done for them. Please see Table 3 for statistical details.

**TABLE 3:** Intergenerational differences in parenting goals and practices reported by mothers.

	*N*	*p*
General goals		
Grandmothers’ goals more collectivistic, mothers’ goals more individualistic	20	
Grandmothers’ goals more individualistic, mothers’ goals more collectivistic	10	<0.001
Specific goals		
Grandmother wants child to be happy	6	0.0178
Mother wants child to be happy	16	
Grandmother wants child to be a regular person	4	0.0401
Mother wants child to be a regular person	12	
Grandmother wants child to have freedom to choose a satisfying career	2	0.0081
Mother wants child to have freedom to choose a satisfying career	12	
Academics		
Achievement more important to grandmother	6	<0.001
Achievement more important to mother	24	
Grades and class ranking more important to grandmother	10	0.0014
Grades and class ranking more important to mother	17	
Grandmother provided more academic guidance and control	6	<0.001
Mother provided more academic guidance and control	23	
Extracurricular courses		
Mothers had more	0	<0.001
Children have more	30	
Children’s happiness		
More important to grandmothers	2	<0.001
More important to mothers	28	
Children’s social adjustment		
More important to grandmothers	0	<0.001
More important to mothers	30	
Conflict between academic competitiveness and socioemotional well-being		
Mother perceives child has or had this conflict	25	<0.001
Mother does not perceive child has or had this conflict	4	
Conflict greater for mothers	1	<0.001
Conflict greater for children	29	
Health/well-being more important for children than academic achievement		
Grandmothers	15/30	<0.001
Mothers	30/30	

*Whereas the N in Table 2 is 60 because respondents reported separate information about each generation, the maximum N in Table 3 is 30 in most cases because, for each variable, each of the 30 mothers was asked to make one comparative judgment concerning generational differences. In all variables except for Specific goals, an N of less than 30 indicates missing data. Except for specific goals, binomial tests assessed whether respondents experienced one direction of generational difference significantly more often than would be expected by chance. The chance level was considered to be a situation where generational differences occurred equally frequently in both directions.*

*For the last variable, a separate judgment was made for the mothers and grandmothers, so the N is 60.*

*For specific goals, participants could list as many or as many or as few goals as they wished. Therefore binomial tests were done separately for each goal, and the total N for all specific goals together is more than 30.*

*Each binomial test assesses whether one generation was associated with a particular childrearing goal more frequently than the other. The chance level for each goal was considered to be equal number of mentions for both generations.*

Mothers also talked about their children’s daily schedule after school, and all 30 mothers reported that their children had taken more extracurricular courses compared to them during their childhood (see Table 3 for statistical details). Most children right now were taking at least one course, while most mothers had never taken any extracurricular courses when they were growing up. Twenty-six out of 30 mothers mentioned that their children were taking English classes, and 23 out of 30 mothers mentioned that their children were taking mathematics classes. Most mothers thought that taking extra tutoring classes after school could be effective in further improving their children’s academics and helping them get into a good middle school. The arts were also very popular among the current generation of Chinese children: 10 out of 30 mothers reported that their children were learning to play the piano; 10 of them reported that their children were learning to draw, and 10 of them reported that their children were learning to dance.

**Hypothesis 8:** *Today’s mothers of young children would impose more parental guidance and control on their fifth-grade children’s academic work and activities.* Twenty-three out of 29 participant mothers felt that they imposed significantly more guidance and control on their children’s academic work than their own mothers had done with respect to their own academic work when they were children (see Table 3 for statistical details).

**Hypothesis 9:** *Today’s mothers of young children will perceive that they are more concerned about their children’s psychological and social well-being than was the case for their mothers.* For the current generation of mothers, the most frequent child rearing goal was for their child to be “happy” (*N* = 16); only six mothers felt their own parents wanted them to be “happy.” The next most frequent goals for the current generation of mothers were for their child to be “a regular person” or to have the “freedom to choose a satisfying career” (*N* = 12 each). Their perceptions of their own mothers’ goals were quite different: only four mothers felt a parental expectation of being “a regular person;” and only two felt that their parents wanted them to have “freedom to choose a satisfying career.” (see Table 3 for statistical details).

Twenty-eight out of 30 participant mothers perceived their children’s happiness to be more important to them than their own happiness had been to their mothers. All 30 mothers perceived their children’s social adjustment to be more important to them than their social adjustment had been to their mothers (see Table 3 for statistical details).

### Comparing Conflict Across the Generations

**Hypothesis 10**: *Today’s mothers of young children will perceive themselves as experiencing more conflict, compared with their own mothers, between two developmental goals for their children: academic competitiveness and social-emotional well-being.* An overwhelming majority (25 out of 29) participating mothers perceived their children had experienced or were undergoing conflicts between achieving academic competitiveness and maintaining socioemotional well-being. Sadly, our implicit conflict measure showed that 29 out of 30 mothers of the second generation also felt that their children experienced this kind of conflict significantly more than they had experienced it during their own elementary school years (see Table 3 for statistical details).

While both kinds of goals – academic achievement and health/well-being – were perceived as becoming more important over time, there was also an historical shift in the balance of the two goals. All 30 mothers expressed that health and well-being of their children was equally or more important than children’s academic achievement. Only half this number thought that their mothers had felt that their health and well-being was equally or more important than their academic achievement (see Table 3 for statistical details).

### How Mothers Experience Shifting Developmental Goals and Navigate Conflict Between Them: Intergenerational Case Studies

The following qualitative analysis focuses on intergenerational shifts within individual families. Our case studies begin by documenting intergenerational shifts in children’s activities and responsibilities; these data are relevant to the last hypothesis. The case studies go on to illustrate the intergenerational increase in perceived importance of academic achievement and socioemotional well-being. They then document mothers’ experience of conflicts between balancing academic achievement and socioemotional well-being. Lastly, the case studies reveal how different mothers navigate these conflicts.

In each of the intergenerational family case studies that follow, the quotes are sequenced to first show mothers’ perceptions of their parents’ behaviors and attitudes, followed by reports of their own parenting behaviors and attitudes. Note that the inclusion of education level and number of siblings for both mother and grandmother documents on the level of individual families the intergenerational sociodemographic changes that accompany changing parental goals.

Brackets indicate words that have been added by the translator for clarity. Dots indicate deletions of less relevant material in the interview.

After the results relevant to Hypothesis 11, which are entirely qualitative, the other intergenerational case studies all illustrate the dominant (and statistically significant) historical trends reported earlier.

**Hypothesis 11**: *In adapting to more Gesellschaft environments in the current generation (urbanization, more technology, greater economic resources) children will lose activities that contribute to family subsistence; these tasks will be replaced by school-related activities such as homework.*

#### Intergenerational Differences in Children’s Responsibility for Household Chores in Mother’s Rural Environment and Child’s Urban Environment

Mother #17 (b.1969, four siblings, her mother’s education: no formal education)

*We lived in the rural area. At that time, when we finished our homework and got off school, we would be doing chores*…. *We would go out into agricultural fields, and I would clear out the weeds. At that time, we would also feed the chickens and collect ragweed for the pigs, helping out with our family, all of this stuff.*

Child (b.2003, one sibling, mother’s education: high school)

*My kid does some chores right now. During school time, she washes dinner dishes. She does not have time for other things since she has to go to school. During this summer vacation, I would like her to learn how to cook, so she started cooking these days*. *Sometimes during vacations, she would wash her underwear and socks. She would not wash them during school time. I did much more than my child is doing right now. What she does right now counts as nothing.*

#### Intergenerational Differences in Children’s Responsibility for Household Chores: Mother Who Spent the First 2 or 3 Years in Rural Area, but Grew Up in the City

Mother #30 (b.1973, one sibling, her mother’s education: middle school)

*During my childhood, I did not spend my time after school doing homework; I was doing household chores. At that time, because our parents were at work, and we usually got off school early, before 5 PM, before they got off work at 6, we would need to wash the ingredients, which were already put in the basket. At that time, not every unit had tap water, it was not that advanced. The whole floor would need to share two [rooms], one on the left and one on the right*. *We called it a sink room. When you entered the room, on the left and right were 10 to 20 taps each, and everyone would be there to wash things, vegetables, clothes, everything that needed to be cleaned was cleaned there. So the advantage of a household with children was that you would not need to [wash] after work. When we grew older and started elementary school, we had learned about how to wash stuff; we would wash the vegetables leaf by leaf, meat, ingredients, We did know how to wash things. After washing, we put them into the basket and waited for them to dry. Then we made rice, it was very easy: using the rice cooker, you wash the rice, [parents] would tell you about the amount of water to put, it was the same for every meal every day for four people. Then you plugged it in, and when our parents came home, they just needed to cook. I would help my parents with what I could. If the floor was dirty, I would sweep the floor. I also have a younger brother, I would need to take care of him, take him home from the kindergarten, stuff like that.*

Child (b.2004, only child, mother’s education: associate degree/college degree)

… *Yes, I have asked my child to do household chores when he was quite young. First, we started to train him to take a shower by himself since 4 or 5 years of age*. *Gradually after a couple of months or even 1 or 2 years, we found that he could do a great job taking a shower, and he was more independent in this area compared to his peers. Starting from Grade two or three, he would need to do household chores: daily tasks would include cleaning shoes, watering the flowers, feeding the fish, changing the water in the fish tank, he would also do that, and then dumping the trash, sweeping the floor, it is a must, making tea for his father, anyway we ask him to do no fewer than six chores per day.*…*He always complains about it, he said, I have known a lot of students in my class, they do not have such requirements at home. Somebody would only wash his own bowl. My child would need to wash his own bowls and plates, but he would also need to do chores for the family*. *I said it is different, every parent has different requirements for their children. I did many more chores during my childhood. The living standard at that time was not like today*. *We had no washing machines, no electrical appliances, no washers, no fridges, no TVs, no phones, nothing. You would need to do many things by hand, unlike now, for laundry [you] only need to throw [your] clothes into the washer*.

#### Intergenerational Differences in Children’s Responsibility for Household Chores: Mother Who Was Born in and Grew Up Entirely in the City

Mother #2 (b.1970, one sibling, her mother’s education: elementary school)

“*[My] parents also did not require me to come home at a certain time, [so I] always played around near my home. After 6PM and [if my] parents did not come back, [I] would go home and make dinner, cooking some noodles for myself to eat. My parents were very busy at work. [I] would do laundry and cook by myself, stuff like this. Sometimes I saw [my] parents very tired, I would stay at home cleaning the floor and the windows, I would do quite a lot of household chores.”*

Child (b.2004, only child, mother’s education: associate degree/college degree)

*“Yes*, *my child does relatively few household chores. She seldom, once per week, or once per month, half a year, she would say, let me help you wash the dishes, I would say alright. [She is] unlike us doing [household chores] every day. She is quite busy with her studies, much busier than we were; we were not that busy with school, [we] helped parents with household chores more.”*

*Summary of case studies showing intergenerational differences in children’s responsibility for household chore*s. In the first case study, we can see a difference in children’s responsibility for household chores between generations. This mother lived in the rural area during her childhood, and she was assigned certain jobs; her work was viewed as obligatory and necessary to support her family. She is typical of her generation, as, at her time, children doing chores was an indispensable part of family contribution in rural communities. On the other hand, this mother attributes her child’s minimal involvement in household chores to going to school.

In the second case study, the mother, whose parents were clearly from a rural area, was in charge of essential chores during her childhood in the city, such as preparing for dinner and taking care of younger siblings. As in the first case of rural-urban migration, children doing chores was an indispensable and obligatory part of family contribution when the mother was growing up. She attributes the decrease in children’s responsibilities for household chores between the two generations to an improvement in living standards, especially the widespread use of electrical appliances such as washing machines, which have freed people from doing some chores by hand. On the one hand, despite her son’s strong disagreement, this mother also requires him to complete at least six chores per day as both a form of family contribution and a way to establish child independence. Given the individualistic turn of society, it is not surprising that she sees children’s engagement in household chores as a way to foster independence and train a sense of personal responsibility. It is also clear from her son’s observations about his classmates that a chore requirement is no longer normative in the urban environment.

The third case study, is similar to the second one. This mother, despite being born and growing up in an urban area, still did some household chores for her family on a daily basis, such as making dinner for herself, doing laundry, and cleaning the house. Importantly, she experienced her work as self-initiated, work that served as an important help to her parents. Compared to her childhood, her child does many fewer chores and does them less frequently. She attributes this intergenerational decline in children’s responsibilities for household chores to her child’s busy studying schedule.

#### Perceived Intergenerational Increase in Importance of Grades and Academic Competitiveness

***Case 1: Mother #20, was coded as caring more for the academic achievement of her child than her own mother had.***

Mother (b.1971, four siblings, her mother had no formal education)

*My parents at that time had never said they had any requirements for us because we always managed ourselves. Yes, they did not need to worry at all. We relied on ourselves to finish homework, and my parents never asked me to get a certain grade or a certain place during exams. Because at that time, my parents did not get any education, so they had no requirements for us, but we tried our best at school.*

Child (b.2004, only child, mother’s education: high school)

*Compared to my parents, I have more expectations of my daughter’s academics. [My child’s academics] are pretty important, and we feel quite under pressure as well. Take a look at our society right now – there is a lot of competition. If she really fails at her academics or something, to be honest, it will be pretty hard for her to find a job. It is terrible, so I pay much attention to her academics.*

***Case 2: Mother #30, was coded as caring more for the academic achievement of her child than her own mother had.***

Mother (b.1973, one sibling, her mother’s education: middle school)

*In my memory, [my parents] did not pay attention [to my academics], but they would ask about my final exam grades. Then they would only, they would not judge, they would only say, if you did poorly*… *they would say, you would need to work hard in the next semester. If you did very well, they would say keep it up.*…. *in my memory, it was only limited to my finals’ weeks, the final examinations.*

Child (b.2004, only child, mother’s education: associate degree/college degree)

*Compared to me right now, I pay much more attention [to my child’s academics] than my parents did.*

***Summary of cases showing perceived intergenerational increase in importance of grades and academic competitiveness***. Both cases exemplify the perception in the sample as a whole that the current generation of mothers is more involved in and more concerned about children’s current and future academic achievement than the prior generation of mothers was. In both Case 1 and Case 2, this trend of increased parental care about children’s academic achievement is correlated with a difference in education levels between the two generations, as well as smaller family size.

#### Perceived Intergenerational Increase in Parental Academic Guidance and Involvement

***Case 1: Mother #22, was coded as providing more parental control than her mother had.***

Mother (b.1977, one sibling, her mother’s education: middle school)

*Compared to myself, my parents had received much less education. I had started elementary school at a fairly young age. I was also the oldest among my siblings, so I had been a relatively thoughtful kid. So, at that time, what my parents provided me with my academics, either tutoring or expectation, was not much, not much. I depended on my self-discipline much more.*

Child (b.2004, only child, mother’s education: master’s degree)

*Right now is different. Although my daughter has a pretty good learning ability, but since she entered kindergarten, I have been checking her homework assignments every day: first, I want to see whether she finishes it; second, I want to know how she masters what she learns at school that day through her homework performance*. *Now my daughter is entering sixth grade and will soon get into a middle school*. *Starting this January during winter break, I signed her up for tutoring classes to improve her grades.*

***Case 2: Mother #10, was coded as providing more parental control than her mother had.***

Mother (b.1975, two siblings, her mother’s education: elementary school)

*[My parents] didn’t care what I had learned. They even didn’t know how many classes I was taking or how much my teacher had taught me.*

Child (b.2003, only child, mother’s education: bachelor’s degree)

*[I] definitely care more about my child than my parents did about us. Because a child is like, a kid, you have to tell him: this is important; you have to pay attention to this; you see you always make mistakes here; you need to think this way, not that way. If nobody tells him [that], he will not have known*. *I know clearly what classes are important. It is because of my miserable experiences that I know children now need help.*

***Summary of cases showing intergenerational increase in parental academic guidance and involvement***. Both mothers see their own mothers as imposing less academic guidance or control compared to what they are now doing with their own children The greater involvement of current mothers in their children’s academics is perceived as both a result of and a solution to their increased concern about their children’s academic competitiveness and personal success. Their increased education, relative to their mothers’, makes the increased academic guidance possible. In each case, family size is smaller in the current generation.

#### Perceived Intergenerational Increase in Mothers’ Concern About Their Children’s Psychological Well-Being

***Case 1: Mother #12, coded as caring more about her child’s happiness than her mother had cared about hers.***

Mother (b.1977, two siblings, her mother’s education: elementary school)

*They seemed to pay no attention, none*…. *They did not even check with me. You would need to rely on yourself for all of this stuff, you would need to adjust your mood and self-process all this information.*…. *They just wanted us to eat well, not get sick, and*… *get home after school, they would be alright with all these.*…. *Even for my first period, my mother did not teach me anything.*

Child (b.2004, one sibling, mother’s education: associate degree)

…. *I think it is pretty important, because sometimes when I observe my child, I feel like she is having some problems with her emotional well-being more or less. Sometimes I ask her what happy things have happened at school and whether she has had any argument with her classmates. Sometimes*. *I just observe her. Sometimes when she was happy, she would say things about herself, but when she grew older, she basically stopped talking about school stuff*. *I sometimes would think about this problem myself.*

***Case 2: Mother #14, coded as caring more about her child’s happiness than her mother had cared about hers.***

Mother (b.1976, four siblings, her mother had no formal education)

*No, they definitely did not pay attention to [my happiness]*. *For some stuff, we might talk to our older sisters, but we did not talk to our parents. At that time, I feel like we seldom communicated with our parents: they said they were out for work; when they arrived home, they would be busy doing household chores. For us, we would play outside. After playing, we would go back, finish our homework, take a shower and go to bed. We rarely communicated with each other*. *It is a different generation.*

Child (b.2004, only child, mother’s education: secondary school)

*It is definitely very important.*…. *I also worry about her a lot, like accidents, so when I talk to her, I would not say extreme words.*…. *I once, when she was little*,…. *at that time, I was still working, I was very busy and tired. I talked to her, I said you should listen to my words, do not always make me angry. If you make your mother angry, I will grow old. I would talk to her like this. Then she suddenly (said), let me tell you a secret, mommy*. *If I were dead, I would not [be able to] make you angry, and you would not grow old. Ah I was so frightened*; *she was so little, yet she said things like that, like that. Why some kids, they would jump off the buildings when they are criticized by their parents, there were instances like this. She did talk to me like this, and I said, don’t you ever think like that. I tried to guide her carefully*. *Kids right now are so vulnerable, so vulnerable, yes.*

***Summary of cases showing perceived intergenerational increase in mothers’ concern about their children’s psychological well-being***. These mothers characterize the sample: they perceive their own mothers as paying little or no attention to their emotional well-being, while they focus a lot on their child’s happiness and mental health. Besides a decrease in family size, this second mother also attributes this increase in concerns for children’s happiness to an increase in mental health problems and suicide rates among young children in China right now. She lists examples of children who committed suicide after being criticized by their parents and talks about one instance where her own daughter talked about dying. She concludes that current children are very vulnerable, and this heightened vulnerability naturally requires extra inputs of parental attention and changes in actions such as refraining from using extreme words and trying to guide her child carefully. In the case of both families, family size is smaller and education is higher in the second generation.

#### Perceived Intergenerational Increase in Mothers’ Concern About Their Children’s Social Well-Being

***Case 1: Mother #19, coded as caring more about her child’s social adjustment than her mother had cared about hers.***

Mother (b.1971, one sibling, her mother’s education: middle school)

*They did not pay much attention [to my social well-being], and I deeply felt it this way, because since I was very young, my parents rarely cared about my emotional aspects.*… *I*… *did not like to talk to people.*

Child (b.2004, only child, mother’s education: high school)

*I think it is quite important for him to hang out with his friends. I think not only is it a part of personal manners, but how he deals with stuff, how he treats people and things is also important.*…. *Most of the time, my child is a bit introverted, he is not that enthusiastic with people. But I often urge him, I would say if people come to your home, or people come to you, you must treat them with enthusiasm.*

***Case 2: Mother #11, coded as caring more about her child’s social adjustment than her mother had cared about hers.***

Mother (b.1978, two siblings, her mother’s education: elementary school)

*They did not, did not quite pay special attention to whether you were introverted or not. But for my parents, since I was little, they have been teaching me about one thing – a thing that also influences me a lot – that*. *you have to be polite to other people, and this is the most basic principle*. *They have been teaching me [about how to interact with people, with adults, friends and classmates], but they did not pay special attention to this area, no.*

Child (b.2004, one sibling, mother’s education: bachelor’s degree)

*But for us, I think I am asking for more, relatively more for my child in this area compared to what my parents asked for. I think how my child is doing for her academics is the second, as long as her being, something that is going to benefit her whole life*…. *I think it’s important, this area is very important, and my child is lacking such skills.*… *I worry about her social skills the most right now, she is quite introverted, rarely talks*…. *It is going to impact her whole life. I have been in this place, and I think this area is relatively important. I have requirements for her, about people and things. I have asked her to be polite to people, even if you are having a bad mood or your emotions are influenced, you should not lose your temper upon other people. You need to interact with your classmates, you should not be on your own, which will make your world closed and limited*….

*Summary of cases showing perceived intergenerational increase in mothers’ concern about their children’s social well-being.* As these two mothers exemplify, the current generation of mothers is more likely to view their children’s social skills as an essential factor in current society. Goals have also changed. Although both Mother #11 and her parents have taught their children how to interact with other people, her parents focused more on being polite and maintaining harmonious interpersonal relationships; these are considered essential to the community and important in a collectivistic environment. Note that both mothers are critical of their child’s introversion and wish for more extraversion, an historical value change that has occurred in Chinese society as a whole (Chen et al., 1998). In the case of both families, family size is smaller and education is higher in the second generation.

#### Intergenerational Increase in Mothers’ Experience of Conflict Between Two Developmental Goals for Their Children: Academic Competitiveness and Socioemotional Well-Being

(Recall that this variable could be assessed only for the present generation of children.)

***Case 1: Mother #19, coded as perceiving her child is experiencing conflicts between achieving academic competitiveness and maintaining socioemotional well-bein*** *g.*

Child (b.2004, only child, mother’s education: high school)

…. *He sometimes would have conflicts, usually during the time before exams. His father would prepare a lot of practice exams for him to do, and he would be very annoyed and reluctant*. *He was not sure about whether he could get good grades. Then he would start working on the questions very unwillingly. There were a few circumstances I knew that he really did not want to do it.*

***Case 2: Mother #10, coded as perceiving her child is experiencing conflicts between achieving academic competitiveness and maintaining socioemotional well-being***

Child (b.2003, only child, mother’s education: bachelor’s degree)

*Yes, [he would have conflicts], but this pressure is not coming from me, or coming from anyone; it comes from our exam-oriented education system. [We] also have no solution to this kind of conflict. You can only learn well, and if you do poorly in an exam, you can do nothing but try again.*…

***Summary of mothers’ experience of conflict between two developmental goals.*** Here, we can see that these mothers think that their children are experiencing conflicts between achieving academic competitiveness and maintaining healthy socioemotional well-being. These mothers are typical of their generation of mothers, as both find it hard to balance the goal of being academically competitive with maintaining socioemotional well-being. However, some mothers actively tried, as in the next three cases.

#### Mothers’ Strategies for Navigating Conflict Between Developmental Goals: Attempts to Balance Conflicting Goals of Academic Competitiveness and Socioemotional Well-Being

***Case 1: Mother #4, coded as perceiving her child is experiencing conflicts between achieving academic competitiveness and maintaining socioemotional well-being.***

Child (b.2005, one sibling, mother’s education: bachelor’s degree)

*Yes, but if my child runs into conflicts and I see him having very negative emotions, I let him stop doing extracurricular homework. We focus on his emotions, and it is enough that we only finish his school assignments.*

***Case 2: Mother #12, coded as perceiving her child is experiencing conflicts between achieving academic competitiveness and maintaining socioemotional well-being.***

Child (b.2004, one sibling, mother’s education: associate degree)

*She sometimes would have conflicts at least once or twice per week, and my solution to this problem can only be imposing more pressure*. *I would explain to her about her school assignments. I would ask her whether she wants to have good grades. She would say yes. But if she wants good grades, she would need to work hard.*

***Case 3: Mother #13, coded as perceiving her child is experiencing conflicts between achieving academic competitiveness and maintaining socioemotional well-being.***

Child (b.2004, only child, mother’s education: bachelor’s degree)

…. *If this situation happens and she is having conflicts, I definitely support her. If she says she really feels painful and tired, I think for this period of time, she can take some rest*. *I think her mental health is more important than her academic work. If she is having major psychological distress, I think it is alright for her to stop*. *I can even let [her] take a gap year.”*

***Summary of instances where mother attempts to balance conflicting goals of academic competitiveness and socioemotional well-being***. The above three cases provide examples of three strategies mothers reported using when dealing with conflicts between their children’s academic competitiveness and socioemotional well-being. Here, we can see the divergence among responses: Mother #13 mentioned that if her child was having psychological distress due to her schoolwork, she would be willing to let her child stop her academic work or even take a gap year. She thinks her child’s mental health is more important than her academics. Mother #12 confessed that her only solution to these conflicts was imposing more parental pressure. She would try to convince her child to complete her work by lecturing her that only hard work could bring good grades and external compliments. Finally, mother #4 stated that if her child was responding negatively toward schoolwork, she would explain to him the necessity to finish his school assignments, while letting him stop working on extracurricular assignment.

## Discussion

All the sociodemographic differences between the generations reflected the shifts going on in China as a whole. Urban residence and formal education increased significantly across the two generations. Families became significantly smaller. Mothers’ work became more professional. The small increase in divorce across the generations in our samples was similar to the increase in the country as a whole. These changes not only reflect social change in Chinese society as a whole (e.g., Zeng and Greenfield, 2015); they also reflect our prior research on family change in China: family size decreased, and a number of mothers had made a transition from rural to urban residence (Zhou et al., 2017). In accord with Greenfield’s (2009) theory and current research (Huang et al., in prep.^1^), we see all of these sociodemographic factors – more education, smaller families, mothers’ professional careers, and urbanization – as acting synergistically to produce the observed intergenerational shifts in parenting goals. The ecological niche of parenting had changed (Harkness and Super, 1992).

Our qualitative analysis points to the rural-urban shift, augmented economic level, and more technology as factors contributing to children’s lesser responsibilities for contributing to essential household chores. The intergenerational decline of children’s meaningful contributions to the household occurs as parental preference for schoolwork increases. This intergenerational shift draws attention to the decline of children’s collectivistic activities (fulfilling family responsibilities) and the subsequent rise in children’ individualistic activities supported by their parents (focusing on schoolwork). Moreover, we also observed an interesting pattern that some collectivistic activities such as helping the family are now intended as independence training for contemporary Chinese children, which further suggests a change in child rearing goals. Mothers saw that Chinese society had shifted and different developmental goals had become relevant: for example, Mother #2 said “I think [academic competitiveness] should be quite important, because children will graduate in the future and if they do not have this ability to compete, entering the society after graduation will also be quite like that.”

Indeed, both qualitative and quantitative data confirmed the hypotheses concerning parenting goals: today’s Chinese mothers see themselves as having more individualistic and less collectivistic goals than their mothers. They perceive both academic competitiveness and well-being of their children as more important to them than it was to their own mothers. Today’s mothers also perceive their children as experiencing more conflicts than they did between the two developmental goals: academic competitiveness and social-emotional well-being. Way et al. (2013) had observed that current Chinese parents face the challenge of balancing these two goals. Our study shows that this challenge is a generational shift. Zhou et al. (2017) had found perception of a generational shifts in parenting young children in China, with praise becoming more frequent and criticism less frequent. This change could be seen as a sign, earlier in child development, of the greater concern with psychological well-being found in the present study.

These shifts flow from sociodemographic changes in China, like the one-child policy and the urban, market-oriented ecology, which then promotes individualistic values (Zeng and Greenfield, 2015). In line with findings in other parts of the world, we have found that social change in the Gesellschaft direction produces changes in parenting values and practices (Manago and Greenfield, 2011; Weinstock et al., 2014; Garcia et al., 2017). Often it is a rise in parent education that produces altered childrearing values (Abu Aleon et al., 2019). Our case studies illustrate this influence: increased educational level of parents across the two generations was correlated with a perceived rise in awareness of psychological needs of children, as well as a perceived rise in the importance of their children’s academic achievement.

The increased concern with academic achievement and competitiveness is adaptive to the fast-growing market economy and competitive environment for employment (Yan, 2009; Bai et al., 2014). The increased demand for academic achievement may prepare children and help them confront the challenges and competition in the economic environment. The increased concern about social skills and well-being is consistent with the finding that traits such as extraversion, self-expression and assertiveness are becoming more desirable in urban China (Chen et al., 2005). By encouraging children to become more outgoing, self-expressive, and assertive, parents prepare their children with the necessary skill sets to survive and succeed in this new market-oriented environment, full of opportunity and competition.

At the same time, the collectivistic practice of controlling children’s academic activities increased across the generations, as predicted. Hence, there was an intergenerational rise in one form of collectivism. This finding confirms the intergenerational increase in parental control reported by Chinese grandmothers for younger children (Zhou et al., 2017). Note though that increased parental control is in the service of the individualistic goal of academic achievement.

The growing awareness of and concern for children’s emotional well-being align with the increased emphasis on children’s social adjustment and subjective well-being in urban China (Steele and Lynch, 2013). In one of the case studies presented above, the mother showed concern over a child’s suicide case that she saw on the news. Several mothers expressed the same concern in their interviews. It is noteworthy that the suicide rate in China has increased at an alarming rate in recent years, especially in adolescents and young adults and in rural regions with a lack of mental health services (Jianlin, 2000; You et al., 2014). This rise in child suicide led mothers to see that increased pressure for achievement needs to be balanced by concern for children’s emotional well-being. Mothers of today’s generation are more aware of the stress and potential risks mental illness for their children and therefore tend to show more care for their children’s emotional well-being.

This study also reveals a rise in conflict between two individualistic developmental goals: academic achievement and social, emotional well-being. Most mothers in the interview expressed their confusion when they talked about the conflicts they were facing. They knew that both goals were important for their children, but it was challenging for them to maintain a good balance between these two goals. Some mothers expressed that it was hard to raise a child in the fast-changing urban Chinese context, and they weren’t sure whether they were doing it in the “right” way. Sometimes they were content about their children adjusting well socially and emotionally, but after comparing their children to other children with seemingly higher academic achievements, they felt anxious that their children might fall behind and wanted to push their children to a higher level of academic achievement. Some mothers said that their perceptions of and expectations about their children’s development are constantly changing, resulting in experimenting with different parenting methods in inconsistent ways.

### Limitations and Future Directions

Participants in this study were recruited from one school in a middle-class urban area of China. Findings would be different if the same study were conducted in rural China, where social change and shifts toward individualism are taking place at a slower rate and are experienced differently. Future studies can examine the rural population and explore rural-urban differences. By including a rural population, the results may elucidate how social changes are experienced in different ecologies and will provide a more complete picture of social change and parenting in modern China.

Perhaps the greatest limitation of the study is the fact that we could not observe mothers’ and grandmothers’ parenting behavior when their children were of comparable age. However, a new study that did observe maternal behavior in two cohorts – and its connection to observed child behavior – confirms our findings and our theoretical focus. Chen et al. (2021) had observational data on the maternal behavior of urban Chinese samples in a laboratory situation at two chronological periods. Mothers were observed interacting with two cohorts of preschool children, the first cohort studied in 1995, the second studied in 2008. In line with our findings of intergenerational change, mothers in their 2008 cohort were significantly more likely to encourage child autonomy than mothers in 1995. Correlatively their preschool children spent more time on autonomous activities than children in the earlier cohort.

Given the absence of behavioral observation, retrospective interviews, such as those used in this study, may be influenced by mothers’ biased and inaccurate memories of their childhood experiences. However, despite the inherent bias in this method, there are several strengths of using retrospective interviews and recruiting only one generation of mothers in this study. First, how individuals experience social change is of great interest in itself. Mothers’ responses, including their biases, reflect their own perceptions and the way in which they construct their subjective experience of social change. Second, perception of parenting goals is an important guide to parenting practice and predicts actual parenting behaviors. Third, mothers were recruited in the study because they have experienced both sides of parenting: receiving parenting during their own childhood and providing parenting to their own children. They are therefore able to make direct comparisons of both facets of the parenting experience.

The conflicts and uncertainty the mothers were experiencing may erode the quality of children’s care, parent-child relationships, and children’s developmental outcomes. These issues should be examined in future studies. While we have explored the conflict between two individualistic childrearing goals – academic achievement and psychological well-being, we have not explored the conflict that parents experience between teaching their children to become “independent agents able to excel in a competitive global economy” (Fong, 2007, p. 86), an individualistic goal, and helping them to mature in a Chinese society that still values obedience and obligation in both family and work environments, two important collectivistic goals. Not only Chinese parents, but also their children experience this conflict and find it both stressful and confusing (Fong, 2007).

We also did not explore the importance of explicitly collectivistic activities that take place in an urban environment, such as children’s participation in activities to help others. It is possible that such collectivistic activities have to some extent replaced the contributions to family maintenance that are more prevalent in rural environments. These are important areas for future research on the implications of rapid social change for learning environments and child development.

In contemporary China, mothers are often the primary caregivers of their children, which made them better informants for the study than fathers. We also know that fathers become more involved with childrearing as mothers shift to working outside the home (Huang et al., in preparation). As mothers’ work becomes more professional in China, witnessing the findings in the present research, it will be interesting in future research to explore this potential intergenerational change in the role of fathers.

## Conclusion

This study of mothers’ perceptions of parenting goals and intergenerational comparisons reflects the rapid social change in urban China and how people respond by creating learning environments for their children. It provides a foundation for future research to explore how these perceptions impact parenting behaviors and children’s developmental outcomes. It also suggests possible comparisons between urban and rural China, in terms of how social change and the rise of individualism may be experienced differently in distinct ecologies.

## Author’s Note

QB is currently at the University of Pennsylvania, Philadelphia, PA, United States. YC is currently at Art Center College of Design, Pasadena, CA, United States. QY is currently at the University of Toronto, Toronto, ON, Canada.

## Data Availability Statement

The datasets generated for this study are available on request to the corresponding authors.

## Ethics Statement

This study involving human participants was reviewed and approved by Institutional Review Board, University of California, Los Angeles. The participants provided their written informed consent to participate in this study.

## Author Contributions

YC conceived of the study. YC and PG designed the study. YC collected the data, and did the preliminary coding and data analysis. QB and QY coded the data, using a revised coding system created in collaboration with PG. QY and QB also did the reliability coding. All authors contributed to the quantitative analysis. QB and QY carried out the qualitative analysis and translated the quotes used in this article from Chinese to English. All authors contributed to the article and approved the submitted version.

## Conflict of Interest

The authors declare that the research was conducted in the absence of any commercial or financial relationships that could be construed as a potential conflict of interest.

## Publisher’s Note

All claims expressed in this article are solely those of the authors and do not necessarily represent those of their affiliated organizations, or those of the publisher, the editors and the reviewers. Any product that may be evaluated in this article, or claim that may be made by its manufacturer, is not guaranteed or endorsed by the publisher.

## Footnotes

1 Huang, V. B., Greenfield, P. M., Zhou, C., and Wu, M. S. (in preparation). Perception of Cross-Generational Differences in Child Behavior and Parent Socialization: a Mixed-method Interview Study with Grandparents in the United States.
