# Supplementary material for: Mothers’ Experience of Social Change and Individualistic Parenting Goals Over Two Generations in Urban China
Source: Front Psychol. 2022 Jan 3;12:487039. doi: 10.3389/fpsyg.2021.487039 (PMC8763011; doi:10.3389/fpsyg.2021.487039)
Supplement: Supplementary file 1 [file Data_Sheet_1.docx]

Appendix. Interview Questions in English & Mandarin Chinese

**Interview Questions**

**问卷问题**

1. What are your goals for your child? (or what do you want your child to be like when he/she grows up?)

你对孩子的目标是什么？（或 你期望你的孩子长大后成为怎么样的人？）

1. Are these similar or different from your mother's goals for you when you were a child? If different, how?

你觉得你对孩子的这些目标和在你小时候你母亲对你的目标一样还是不一样？如何不一样？

1. How important is your child's academic achievement and competitiveness?

你的孩子学业上的成就和竞争力对你来说重要吗？

1. Do you think your academic achievement and competitiveness was more or less important for your mother when you were a child, or was it the same?

你觉得跟你孩子相比，你小时候的学业上的成就和竞争力对你母亲来说有多重要？

1. How important is your child's happiness?

孩子的快乐对你来说有多重要？

1. Do you think your happiness when you were a child was more or less important to your mother, or was it the same?

你觉得跟你孩子相比，你小时候的快乐对你母亲来说有多重要？

1. How important is your child's social adjustment to you?

孩子的社会适应能力对你来说有多重要？

1. Do you think your social adjustment when you were a child was more or less important for your mother, or was it the same?

你觉得跟你孩子相比，你小时候的社会适应能力对你母亲来说重要吗？

1. Do you ever feel that there is a conflict between your child's academic competitiveness and his/her social/emotional well-being? Why? Why not?

你有没有觉得你孩子的学业上的成就和竞争力，和孩子的心理健康（社交和情绪的健康）有没有冲突？为什么？

1. Do you think your mother ever felt that there was a conflict between your academic achievement and your social/emotional well-being? Why? Why not?

你觉得在你小时候，你母亲有没有曾经感觉到你的学业的成就和你的社交和情绪上的健康有冲突？

1. What after school activities does your child engage in?

你的孩子参加了那些课外活动？

1. Who selected the activities? Why?

谁选择的这些活动？为什么要参加这些活动？

1. When you were about the age of your child, what after-school activities did you engage in? Who selected the activities? Why?

当你在你孩子差不多大的时候，你参加了哪些课外活动？谁选择的这些活动？为什么要参加这些活动？

1. How do you balance activities that promote academic achievement with those that promote social and emotional well-being? Is this difficult? Why?

你怎么平衡这些帮助孩子学习进步的活动和帮助孩子保持心理健康的活动？你觉得平衡这些活动很困难吗？为什么？

**Demographic Questions**

**基本情况**

1. What is your age?

请问你的年龄是？

1. How old is your child? Does your child have any sibling?

你的孩子多少岁？他/她有兄弟姐妹吗？

1. What is your education level?

你的教育水平是？

1. What is your marital status?

请问你的婚姻状况是什么？

1. What are the education levels of your parents?

你父母的教育水平是什么？

1. Were your parents married when you were a child?

在你小的时候，你的父母的婚姻状态是什么？

1. Are you currently employed? If employed, what is your occupation?

你的工作是什么？

1. **(If married)** Are your husband currently employed? If employed, what is his occupation?

（如果已婚）你先生的工作是什么？

1. What were your parents’ occupations when you were a child?

在你小的时候，你父母的工作是什么？

1. What is the total annual income at your household?

你们家总共的年收入大概是多少？

1. Where were you born and raised? Was that place(s) urban, suburban, or rural?

请问你是在哪里出生和长大的？那里是城市，郊区，还是农村地区？

1. Where was your child born and raised? Was that place(s) urban, suburban or rural?

请问你的孩子是在哪里出生和长大的？那里是城市，郊区，还是农村地区？
